# Data on ecological network projects in Switzerland

**DOI:** 10.1016/j.dib.2025.112169

**Published:** 2025-10-10

**Authors:** Lars Tschus, Franziska Zimmert, Petyo Bonev, Maximilian Meyer

**Affiliations:** a Agroscope, Research Group Managerial Economics in Agriculture, Ettenhausen, Switzerland; b University of St. Gallen, Switzerland

**Keywords:** Agricultural policy, Biodiversity, Switzerland, Agglomeration bonus schemes, Ecological network project, Spatial coordination incentives, Multi-actor oriented agri-environmental schemes

## Abstract

Agriculture is one of the primary drivers of biodiversity loss. One agricultural policy instrument in this context is the spatial coordination of conservation efforts by farmers through ecological network projects, which provide spatially connected conservation areas, such as areas under agri-environmental schemes. However, the success factors for establishing ecological network projects, as well as their effects on various economic and ecological outcomes are still poorly understood. To obtain a deeper understanding of these important questions, we assembled a novel and comprehensive geospatial dataset of network project perimeters at the municipality level in Switzerland. Within these network project perimeters, farmers are eligible to enroll their agricultural plots that need to be managed according to the regulations of agri-environmental schemes. The dataset covers 98 % of Switzerland’s total land surface, including areas with and without ecological network projects. The dataset enables linkage with other data sources for future analyses, such as estimating the effect of network projects on e.g. the take-up of other agri-environmental schemes, farm income and biodiversity. To improve these future analyses, the dataset contains additional information such as the area and start year of an ecological network project, as well as the geographical share of ecological network projects in a municipality.

Specifications Table

**Table:** 

Subject	Agricultural Economics
Specific subject area	Perimeters of ecological network projects in Switzerland at municipality level
Type of data	Geopackage
Data collection	The network project perimeter data were either downloaded from the cantonal GIS databases or requested directly from the cantonal authorities. The municipal areas were obtained from the swisstopo swissBOUNDARIES3D dataset. Geodata of summering areas and lakes were obtained from the agricultural land boundaries from the Federal Office for Agriculture (FOAG). The data were prepared and processed for each canton in RStudio.
Data source location	Zenodo
Data accessibility	Repository name: Zenodo Data identification number: 10.5281/zenodo.15006754 Direct URL to data: https://zenodo.org/records/17248997
Related research article	None

## 1 Value of the Data

• The dataset can answer research questions related to the creation and continuation of ecological network projects.^2^ This knowledge helps scientists and policymakers to identify success factors of such a coordinated agricultural policy.
• The universal mapping identifier (i.e., the municipality) makes the dataset a valuable basis for exploring further areas of research such as the ecological and economic impacts of ecological network projects.
• In that sense, future analyses can shed light on important trade-offs and synergies between economic and ecological goals.
• Our dataset will facilitate comparisons across municipalities or regions to identify best practices and lessons learned for biodiversity conservation.
• Finally, our dataset provides valuable input for regional planning, such as coordinating infrastructure development.

## 2 Background

Agriculture is one of the primary drivers of biodiversity decline, exerting pressure on species worldwide [1]. Currently, >5000 species are directly threatened by agricultural practices, primarily due to extensive deforestation, the expansion of agricultural land, the fragmentation of natural habitats and the intensification of farming activities [2,3]. The urgency of addressing these impacts is underscored by the accelerated rate of species extinction, which is now estimated to be between 100 and 1000 times higher than historical background rates, largely due to anthropogenic factors [4,5].

One approach to mitigating agricultural impacts on biodiversity is ecological network projects, which represent sets of biodiversity-supporting (or conservation) measures that target biodiversity at the landscape (i.e. regional) level. This contrasts with traditional agricultural conservation schemes that target biodiversity at the individual farm level [6,7].^3^ One of the main goals of ecological network projects is to coordinate conservation efforts by farmers in a given region in terms of the choice and location of conservation measures by considering the specifics of the larger landscape [8]. The mechanism of ecological network projects is the coordination of farmers’ conservation efforts through the creation of spatially connected conservation areas, such as areas under agri-environmental schemes. This promotes the genetic and demographic exchange of species, which is of crucial importance because it increases populations’ adaptability and thus contributes to long-term species conservation [[9], [10], [11]].

The spatial coordination of conservation efforts is characterized by different interventions [12], the implementation of which is often examined theoretically (see e.g.,Drechsler [3],Drechsler [13],Wätzold and Drechsler [14]). Empirical applications are rare, partly because few countries have implemented such measures to date (for a systematic review, see Nguyen, Latacz-Lohmann, Hanley, Schilizzi and Iftekhar [15]). In Europe, the Netherlands launched a national program in 2016 [16], and there are also a number of regional agreements in France or the United Kingdom [[17], [18], [19]].

Switzerland is one of the few countries that incentives participation in ecological network projects throughout the country. Participation in ecological network projects is remunerated with so-called agglomeration bonus payments [20]. Farmers who register for these schemes receive fixed monetary compensation for each hectare enrolled in the scheme. The canton determines the payment amount, of which the federal government covers a maximum of 90 percent. However, the federal government also sets maximum amounts for which it will contribute to the financing. In practice, most cantons base the payment amounts on the benchmark defined by the federal government (in most cases 1000 Swiss francs per hectare). The rationale for this subsidy is to compensate for profits foregone from reduced production and for costs from additional labour [20,21]. Each network project implements measures specifically tailored to the needs of regional target and indicator fauna and flora species [22]. These measures build upon a minimum criterion for species protection, which is outlined the direct payments’ ordinance of the Swiss state [21].

Ecological network projects in Switzerland are implemented in two steps. In the first step, an ecological network project is initiated by a stewardship. Stewards can be cantons, municipalities or farmers’ unions, and they are responsible for the project’s long-term maintenance and development [23]. To keep administrative costs low, ecological network projects are usually initiated and managed by municipalities. This means that in most cases, the administrative boundaries of the municipalities correspond to the boundaries of the ecological network project [22]. In this first step, the project’s perimeter and the target species to be protected or promoted are defined. Within the project perimeter, plots enrolled in agri-environmental schemes should be located and managed in such a way as to improve the development and spread of animals and plants. The choice of species is made by either the stewardship or outsourced to an eco-office. Based on this choice, specific protection measures are developed for each target species (e.g. phase-based mowing or the construction of landscape features). In the second step, farmers can decide if they participate and if so, with which plots they participate in the scheme. There is an established documentation procedure for all network projects, with an interim report after four years and a final evaluation of the defined goals after eight years. After eight years, ecological network projects can be extended but all will ultimately end in 2027 due to a policy reform. This policy reform will merge the network project payment and the landscape quality payment to form the new payment for regional biodiversity and landscape quality.

The success factors for establishing ecological network projects, as well as their effects on various economic and ecological outcomes, are still poorly understood. For example, Häusler and Zabel [24] empirically compare the effectiveness of two different spatial coordination rules in a case study for the Swiss canton of Berne. Another related paper examines the factors for participating in an ecological network project in the Swiss mountain region in the canton of Valais [25]. To make it possible for the scientific community to obtain a deeper understanding of these important questions, also covering a larger geographical area, we assembled a novel and comprehensive dataset of network project perimeters^4^ in the whole of Switzerland.

## 3 Data Description

**Fig. 1:**
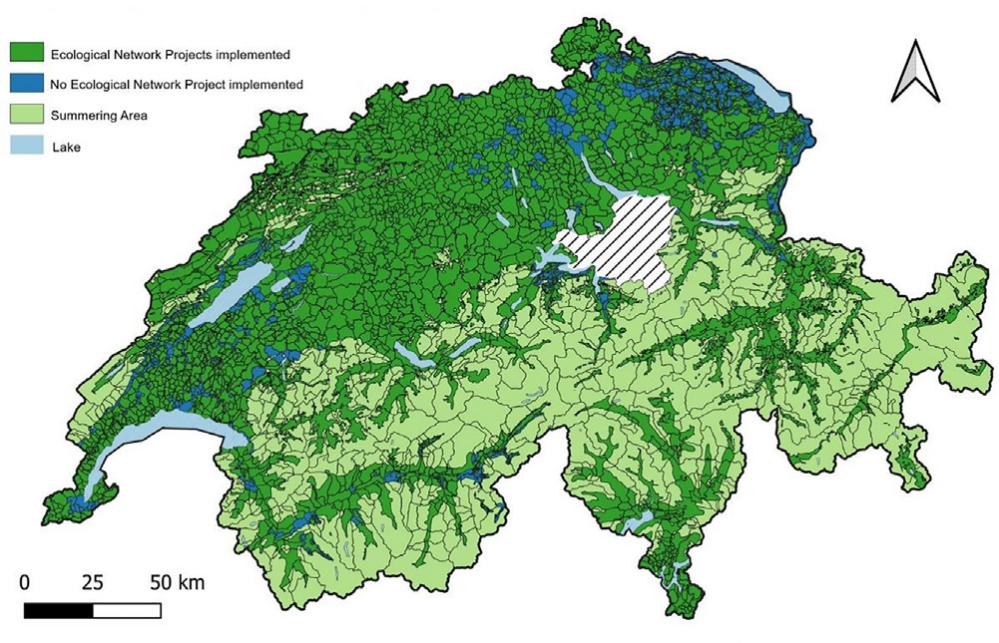
Ecological network projects in Switzerland. The shaded area is Schwyz which is not included in the data set.

The dataset covers all ecological network projects throughout Switzerland, except for those in the canton of Schwyz^5^ (see Fig. 1). The network perimeter of a canton includes all network projects within the cantonal territory. We separated out summering areas and lakes because these areas are not eligible for agglomeration bonus payments.^6^ For administrative simplicity, the boundaries of a network project mostly coincide with the boundaries of one or several municipalities in which the project is initiated [26]. That is why the observational unit is the municipality level in the resulting dataset. The network perimeters are overlaid with the municipality boundaries to obtain the connected areas of the municipality, but it allows us also to identify those parts of a municipality where no network project has been initiated. Providing data at the municipal level also has practical benefits. Additional data that is not geocoded but can be aggregated at the municipal level can be merged with the dataset, enabling a wider range of research questions to be explored. The municipal information can be obtained either via the *comm* variable that relates to the community name or the *bfs_number* variable indicating the community number assigned from the Federal Statistical Office.

The *participation* variable indicates whether an observation has an ecological network project or not. Other values of this variable include “summering area”, “lake” or “overlapping area”. Overlapping areas arise from different underlying datasets and are further explained in Section 4. If only a part of a municipality has an ecological network project, the *part_comm* variable takes on the value “1” and “0” otherwise.

The dataset also contains further information, such as the network project’s name (*title* variable) and the canton it belongs to (*canton* variable). The *start* variable indicates the start year of an ecological network project. If it is extended for another period, the variable still indicates the start year of the first period. In contrast the *start_year* variable indicates the start year of the current period. Similarly, the *end_year* variable indicates the last year of the current period. The stewardship of an ecological network project is indicated by the *owner* variable and can take on the values “cantonal”, “local”, “communal”, “local/communal” or “farmer’s association”. The *project_area* variable gives the size of the area of an ecological network project. In contrast, the *geom_area* variable gives the size of the area of an observation and the *comm_area* variable the size of the municipality area. Additionally, the dataset provides the size of the land where no ecological network project is implemented (*no_ABS_area_per_comm*) or that is categorized as an ecological network project (*ABS_area_per_comm*), summering areas (*summering_area_per_comm*), lakes (*lake_area_per_comm*) or overlapping area (*overlap_area_per_comm*) along with their share of the total municipality area (variable names ending with *_pct*). The *remark* variable includes any additional information (see Table 1 for information on all columns of the dataset and the following section for a more detailed description). Finally, there are manually coded exceptions such as name harmonizations or geometry corrections for which we provide a supplementary table (Table A.2) listing all instances where manual adjustments were made. The result is a dataset showing connected and non-connected areas in Switzerland.

**Table 1:** Description of all columns in the dataset.

Column Name	Explanation
*Title*	Name of the ecological network project if existing. If no ecological network project is implemented in a municipality or in part of it, a missing value (NA) is indicated in this column. The 99 value indicates whether areas were assigned twice in the original network perimeter dataset (which was provided by the cantons). These areas were removed from both network projects and saved as new areas.
*comm*	Municipality name.
*canton*	Abbreviation for the respective canton.
*start*	Indicates the first year of the network project. If a network project was extended for another period, the start date of the first period is included here. Note: Sometimes there were (small) changes to perimeters between two periods as these were merged or extended.
*start_year*	Indicates the start year and the start date of the current period with the current expansion (if available).
*end_year*	Indicates the last year of the current period. In most cases, network projects are extended until 2027, when the new agricultural policy comes into force.
*remark*	Remarks on additional information, such as the current period, extensions to the project duration, name changes of the network projects or similar.
*bfs_number*	The municipality number assigned by the Federal Statistical Office (FSO)*.* Researchers can use this variable as the key for merging with other datasets.
*geom_area*	Size of the area of the observation (in ha).
*owner*	Ownership type: cantonal, local, communal, local/communal or farmers’ association.
*participation*	Indicates whether the observation has an ecological network project (1) or not (0) or is a summering area (2), lake (3) or overlapping area (99).
*project_area*	Total size of the area of the ecological network project (in ha).
*no_ABS_area_per_comm*	Size of the area per municipality where no ecological network project has been implemented (in ha).
*no_ABS_area_per_comm_pct*	Share of area where no ecological network project has been implemented of the total area per municipality (in %).
*ABS_area_per_comm*	Size of the area per municipality where an ecological network project has been implemented (in ha).
*ABS_area_per_comm_pct*	Share of area where an ecological network project has been implemented of the total area per municipality (in %).
*summering_area_per_comm*	Size of the area per municipality considered as summering area (in ha).
*summering_area_per_comm_pct*	Share of summering area of the total area per municipality (in %).
*lake_area_per_comm*	Size of the lake area per municipality (in ha).
*lake_area_per_comm_pct*	Share of lake area of the total area per municipality (in %).
*overlap_area_per_comm*	Size of the area per municipality considered as overlapping area (in ha).
*overlap_area_per_comm_pct*	Share of overlapping area of the total area per municipality (in %).
*comm_area*	Total size of the municipality area (in ha).
*part_comm*	Indicates whether a municipality has an area where only part has an ecological network project implemented (1) or not (0).

## 4 Materials and Methods

To create the dataset, we used two data sources. First, we used the swissBOUNDARIES3D dataset from the Federal Office of Topography (swisstopo) of Switzerland. This dataset includes the boundaries of Switzerland, its cantons and municipalities. However, this dataset contains no information on ecological network projects. Second, we collected data on ecological network projects in the different cantons. These data were either freely available online or requested from the cantonal authorities. Those cantons whose data was not available online have given their consent to publication in a designated contract, which are available upon request from the authors. Table A.1 in the online appendix shows for each canton where the data are available, the cantonal reference, the download link or the email address of the responsible office that provided the data. The different datasets were assembled into a single dataset. The entire data processing was carried out in RStudio and QGIS was used for the visualizations. The R code is provided in a separate file.

In the following section, we describe the data processing steps.

### 4.1 Limiting network project perimeters to one canton

The network project perimeters of a canton often incorporate sections of projects from neighbouring cantons, either due to spatial overlapping or the addition of available data to their own datasets. The cantonal representatives we contacted confirmed that the perimeters within their own boundaries were accurate, but they could not guarantee the accuracy of projects outside their canton. Therefore, we excluded projects located outside the canton of interest. For most datasets, the information to which canton the project belonged was available, which allowed us to remove the network project outside the canton of interest. If this was not the case, the perimeters were imported to QGIS, and the project identifiers for areas outside the canton were written down. These identifiers were then used in RStudio to filter out the external projects from the dataset.

### 4.2 Geometry corrections by network project perimeter

**Fig. 2:**
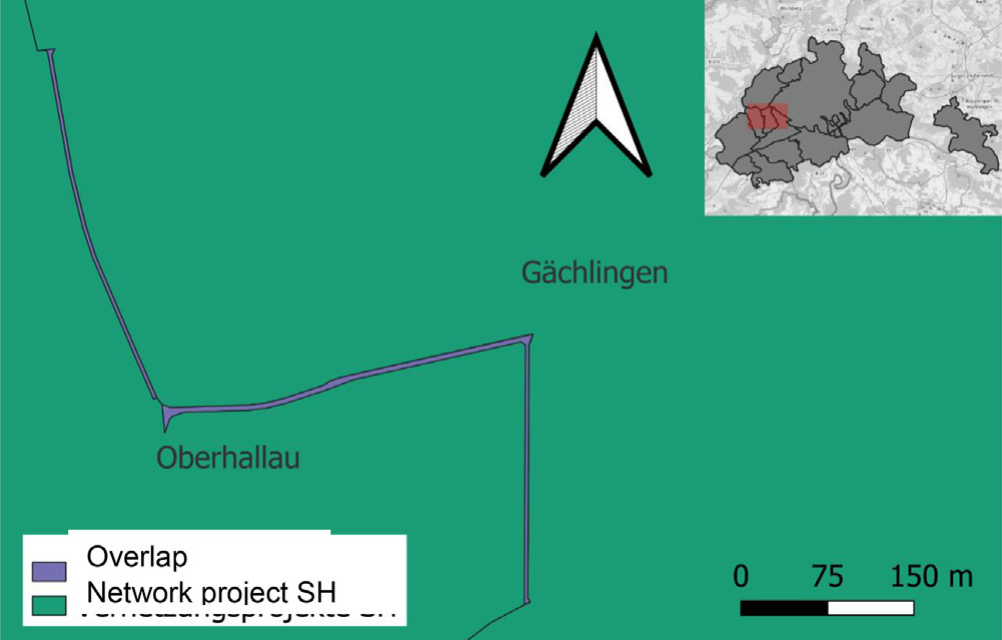
Geometry overlap in the Schaffhausen Canton between the Gächlingen and Oberhallau projects (shown in purple). The red area in the image that is inset at the top right of this figure shows where you can find these exemplary network projects in the Schaffhausen Canton.

We then checked for overlaps between two adjacent network project perimeters. Fig. 2 illustrates such an overlap (in purple) of the network projects Gächlingen and Oberhallau (green). If such areas existed, they were removed from both network projects and highlighted as a geometry overlap in the *title* (99) variable. The corrected dataset was used for further data processing.

### 4.3 Determining network project areas in a municipality

**Fig. 3:**
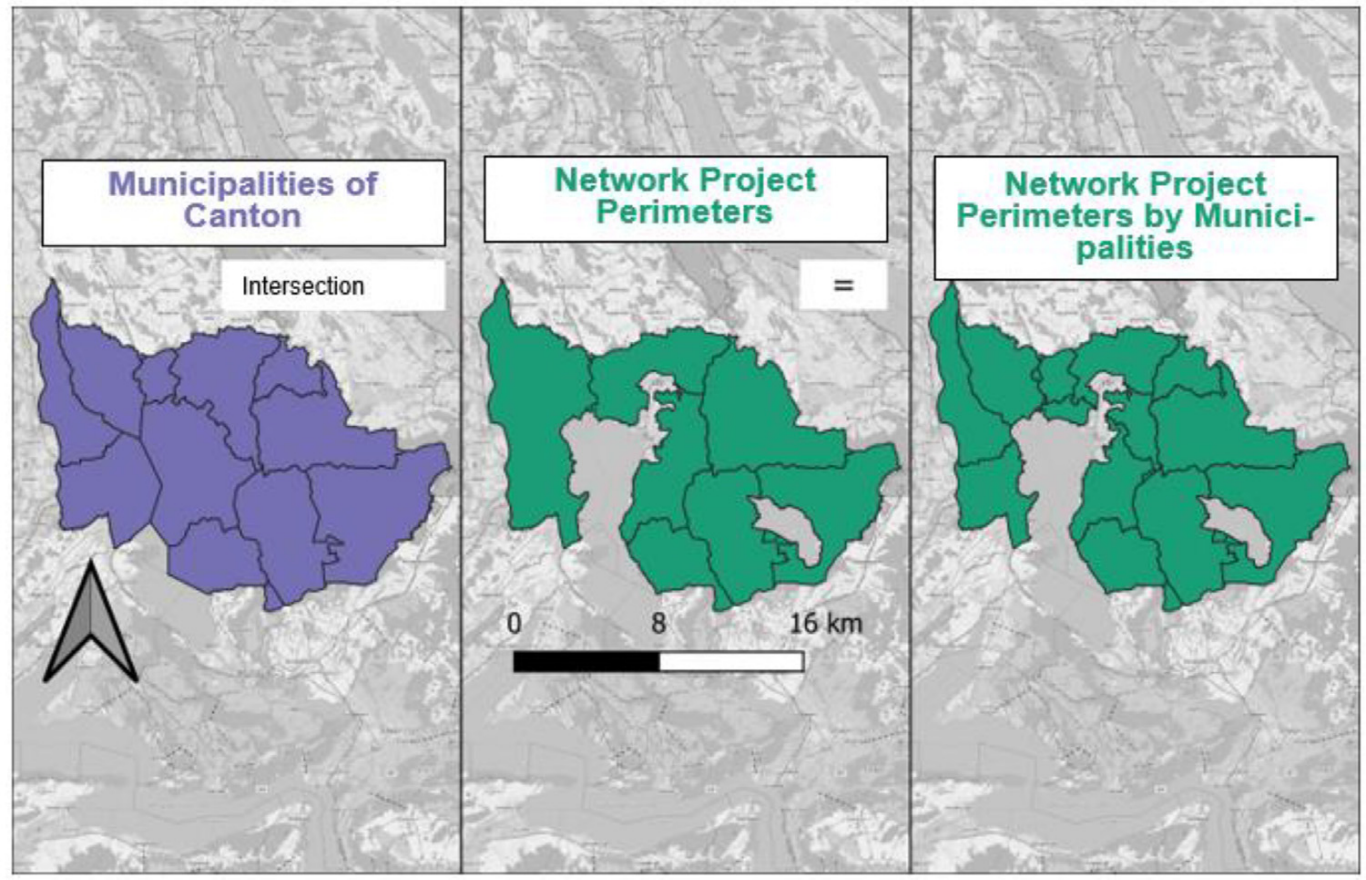
Intersection of municipalities of the Zug Canton (purple) with network project perimeters (green).

After correcting the geometry and filtering the municipalities by canton, the network data were intersected with the municipalities, using *st_intersection* in RStudio. During this intersection, common areas were retained, whereas areas that only occurred in one dataset were excluded. The boundaries (lines around a geometry) of the municipality dataset were transferred to the network dataset and vice versa (see Fig. 3 for an example from Zug Canton). In this way, for each municipality, the areas of network projects were determined.

### 4.4 Determining areas which are not part of a network project in a municipality

**Fig. 4:**
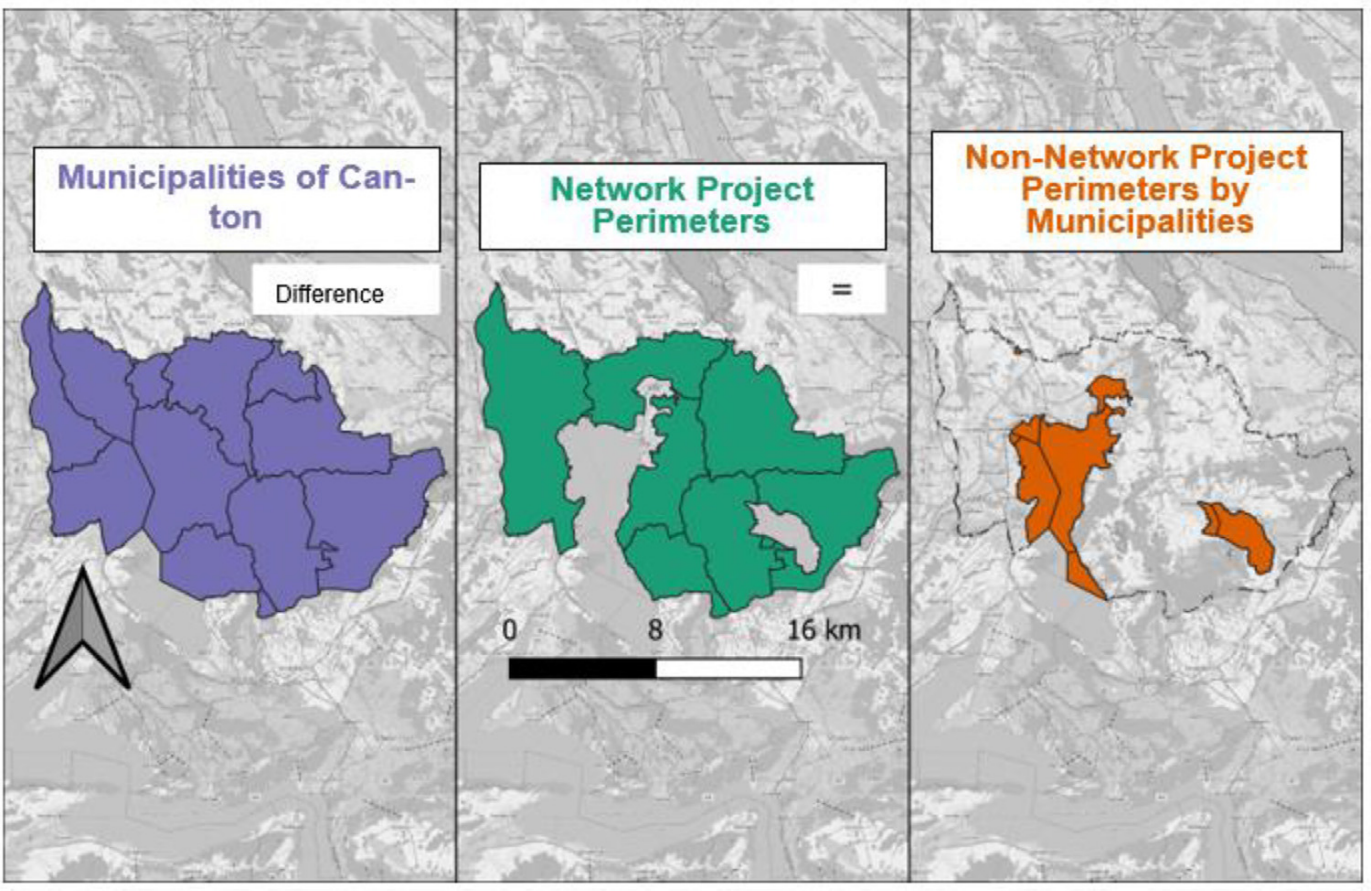
Geometric difference calculation between municipalities (purple) and network project perimeters (green) for Zug Canton. The difference resulted in the creation of a dataset with all areas that are not part of a network project per municipality (shown here in orange).

To obtain the areas where no network project was implemented, we used the municipality data, overlaid it with the network project layer and created the difference, using the *st_difference* function in RStudio. This function calculates the geometric difference (see Fig. 4).

At the cantonal border, there are often small areas that are determined as not having an ecological network project. This is likely because cantons used different boundaries for their network projects—often based on municipal boundaries—than those defined by swissBOUNDARIES3D when establishing the perimeters. Within cantons, this discrepancy in boundaries leads to a similar challenge: Small fractions of a network project may be incorrectly assigned to another municipality. In the final dataset, we kept these small intersections. They can be filtered out by defining a minimum value for the perimeter’s area (*geom_area* variable).

### 4.5 Merging and integrating summering areas and lakes

**Fig. 5:**
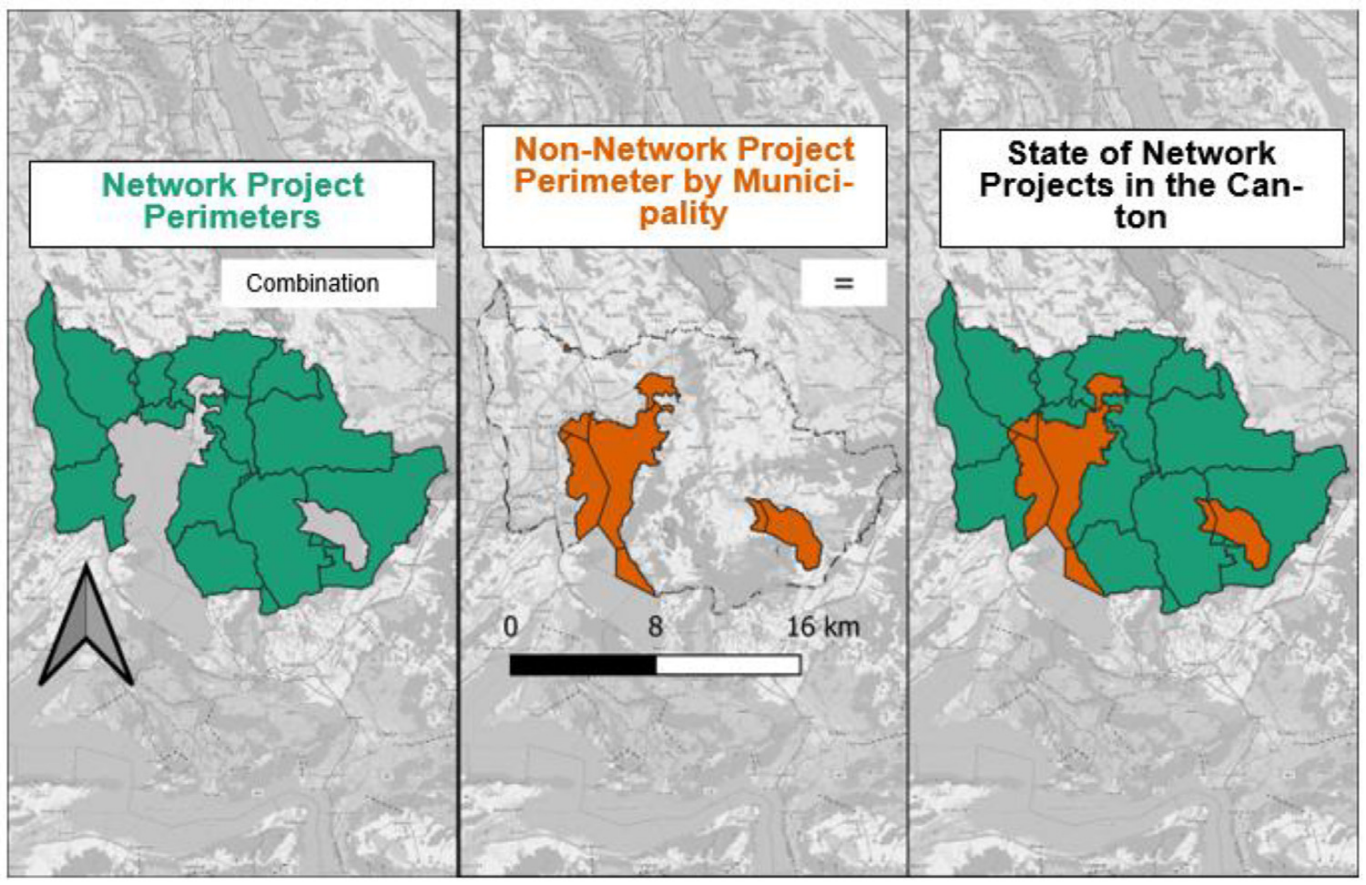
This figure shows the combination of network projects (green) and non-network project perimeters (orange) per municipality for Zug Canton.

After processing the areas belonging to a network project or not for each municipality, they were merged using the *rbind* function of RStudio (see Fig. 5).

After merging, geodata on summering areas and lakes were integrated into the dataset using the agricultural land boundaries from the Federal Office for Agriculture (FOAG). Summering areas were assigned a value of 2 for *participation*, while lakes were assigned a value of 3. If any of these areas had previously been part of a network project, the associated project information (including project name, start and end years) was removed. We filtered out the areas affected by summering areas or lakes from the state of network projects dataset. To enable subsequent merging, we then filtered out the unaffected areas. This was achieved by calculating the geometric difference between the summering areas/lakes dataset and the state of network projects dataset, creating the areas unaffected by summer pastures or lakes.

### 4.6 Calculating different information on network projects

Finally, we calculated further information important for understanding and analysing network projects. As an example, we can see from Table 2 that the Isenthal municipality has an area of about 60 ha labelled as lake and 0.001 ha of overlapping areas.

**Table 2:** Data extract of the exemplary Isenthal municipality.

	Title	Comm	Canton	Start_Year	End_Year	Bfs_Number	Geom_Area	Owner	Participation	Project_Area
1	99	Isenthal	UR	NA	NA	1211	0.001	NA	99	NA
2	Gitschenen	Isenthal	UR	2016	2027	1211	130.069	local	1	130.069
3	Urnersee - West	Isenthal	UR	2018	2025	1211	1152.113	local	1	2927.266
4	NA	Isenthal	UR	NA	NA	1211	4814.336	NA	2	NA
5	NA	Isenthal	UR	NA	NA	1211	60.194	NA	3	NA
6	NA	Isenthal	UR	NA	NA	1211	10.292	NA	0	NA

*Project_area* represents the total area of a network project. It was generated by grouping the dataset by project (*title* column), merging the geometries within each group and recalculating their area. In Table 2, we see that the Gitschenen network project has an area of about 130 ha in the Isenthal municipality. In contrast, 1152 ha of the Urnersee – West project lies within the Isenthal municipality, although the project is 2927 ha in total. Areas outside project boundaries are defined as a missing value. To indicate whether a municipality has implemented a network project on parts of its area, we created a binary variable called *part_comm*. We considered a municipality to be partially connected if >2.88 % of the total community area was part of a network project. This threshold was selected because it is the smallest share of a total municipality to be a network project that we know of for certain, which is the Lupfig municipality in Aargau Canton. For more details, see Fig. A.1 and the description in Section B of the online appendix.

The sizes of the areas for the different participation groups (no agglomeration bonus scheme [ABS], ABS, summering area, lake and overlap) were created by grouping the dataset by municipality and participation status and then calculating the area of the geometry. The share of the area belonging to each participation group of the total municipalty area was then calculated in *no_ABS_area_per_comm_pct, ABS_area_per_comm_pct, summering_area_per_comm_pct, lake_area_per_comm_pct* and *overlap_area_per_comm_pct*. We present a description of all columns in the dataset in Table 1.

## Limitations

Unfortunately, the dataset does not cover the Swiss cantons of Schwyz. In addition, we found discrepancies in the geoinformation of the two underlying datasets, which explains the occurrence of overlapping areas. These observations are included in the final dataset and can be filtered out by researchers themselves. Finally, although the dataset reflects eligibility for participation of farmers in network projects, this does not reflect actual enrollment of farms. However, this data can be obtained from both farm plot perimeters and the farm accountancy data network (FADN), which informs about enrollment at the plot- and at the farm-level, respectively. These data can then be merged with the dataset presented here.

## Ethics Statement

The authors have read and followed the ethical requirements for publication in *Data in Brief* and confirm that the current work does not involve human subjects, animal experiments or any data collected from social media platforms.

## CRediT Author Statement

**Lars Tschus:** Conceptualization, Data Processing, Writing – original draft. **Franziska Zimmert:** Conceptualization, Data Processing, Writing – review & editing. **Petyo Bonev:** Conceptualization, Writing – review & editing. **Maximilian Meyer:** Conceptualization, Data Processing, Writing – original draft, Writing – review & editing.

## Data Availability

ZenodoEcological Network Projects in Switzerland (Original data).

## Appendix Supplementary materials

Image, application 1

## Footnotes

2 Ecological network projects are also called agglomeration bonus schemes (ABSs).

## Footnotes

3 In Switzerland and in the European Union, the main agri-environmental schemes that target biodiversity conservation at the farm level are action-based and result-based direct payment schemes; see [8] for a detailed description.

4 A perimeter is defined as the spatial extent of a given network project.

5 Schwyz is not included as data was not provided by the canton.

6 Summering areas are high-altitude pastures used during the summer months for grazing livestock. As glaciers and rocks are within the summer farming zone, its exclusion automatically removes these non-agricultural areas from the dataset. In contrast, settlement areas are part of the officially defined agricultural zones. Farming frequently occurs within and around settlements, and ecological network projects can extend into urban areas—for example, the city of Winterthur hosts three such projects, including its city center. Excluding settlements would therefore result in a loss of relevant information.
