# Supplementary material for: Data on ecological network projects in Switzerland
Source: Data Brief. 2025 Oct 10;63:112169. doi: 10.1016/j.dib.2025.112169 (PMC12581624; doi:10.1016/j.dib.2025.112169)
Supplement: Supplementary file 1 [file mmc1.docx]

Online Appendix

1. **Tables**

**Table A.1**: Data sources (network perimeters, municipalities and agricultural zone boundaries) used to create the dataset.

| Canton | Available online | Reference | Contact |
| --- | --- | --- | --- |
| AG |  | Kanton Aargau. Perimeter Vernetzungsbeiträge. https://www.ag.ch/de/verwaltung/dfr/geoportal/geodaten/geodatenliste?rewriteRemoteUrl=/details/AGIS.al_vpperimeter?searchcontext%3DVernetzung (accessed 18 November 2024) |  |
| AI |  | Kanton Appenzell Innerrhoden, Land- und Forstwirtschaftsdepartement. Vernetzungsperimeter. | Landwirtschaftsamt AI: info@lfd.ai.ch |
| AR |  | Departement Bau und Volkswirtschaft, Amt für Landwirtschaft. Biodiversitätsförderbeiträge. Botanische Qualität und Vernetzung auf der landwirtschaftlichen Nutzungsfläche. <https://ar.ch/fileadmin/user_upload/Departement_Bau_Volkswirtschaft/Landwirtschaftsamt/Beratung/Broschuere_Biodiversitaetsfoerderbeitraege.pdf> (accessed 18 November, 2024) | Dataset created using information from the given reference with municipal data from swisstopo. |
| BE |  | Amt für Landwirtschaft und Natur des Kantons Bern. Vernetzungsprojekte nach ÖQV. 2024. https://www.agi.dij.be.ch/de/start/geoportal/geodaten/detail.html?type=layer&code=PP (accessed 18 November 2024) |  |
| BL |  | Ebenrain-Zentrum für Landwirtschaft, Natur und Ernährung. Vernetzungsperimeter. | Ebenrain-Zentrum für Landwirtschaft, Natur und Ernährung:  ebenrain@bl.ch |
| BS |  | Ebenrain-Zentrum für Landwirtschaft, Natur und Ernährung. Vernetzungsperimeter. | Ebenrain-Zentrum für Landwirtschaft, Natur und Ernährung:  ebenrain@bl.ch |
| FR |  | Kanton Freiburg, Kompetenzzentrum für Ausbildung, Beratung und Vollzug im Bereich Landwirtschaft “Grangeneuve”. https://geo.fr.ch/OpenData/FGDB/R%C3%A9seaux%20%C3%A9cologiques.zip (accessed 1 April 2024) |  |
| GE |  | DT - Département du territoire, Office cantonal de l’agriculture et de la nature. RESEAUX AGRO-ENVIRONNEMENTAUX (RAE). 2023. https://ge.ch/sitg/sitg_catalog/sitg_donnees?keyword=&geodataid=4457&topic=tous&service=tous&datatype=tous&distribution=tous&sort=auto (accessed 18 November 2024) |  |
| GL |  | Kanton Glarus - Volkswirtschaft und lnneres. Abteilung Landwirtschaft. Vernetzungsperimeter. | Abteilung Landwirtschaft: landwirtschaft@gl.ch |
| GR |  | Kanton Graubünden, Amt für Landwirtschaft und Geoinformation. Vernetzungsperiemter. | Amt für Landwirtschaft und Geoinformation: info@alg.gr.ch |
| JU |  | Canton du Jura, Service de l’Economie rurale. Paiements directs: Reseaux Agro-Environnementaux. | Service de l’économie rurale (ECR): paiements-directs.ecr@jura.ch |
| LU |  | Kanton Luzern, Raum und Wirtschaft (rawi). Vernetzungsprojekte. https://daten.geo.lu.ch/download/vnprojxx_col_v1 (accessed 18 November 2024) |  |
| NE |  | Canton de Neuchâtel, Département du développement territorial et de l’environnement, Service de la faune, des forêts et de la nature. Reseaux Agro-Environnementaux. | Service de la faune, des forêts et de la nature: SFFN@ne.ch |
| NW |  | Kanton Nidwalden, Landwirtschafts- und Umweltdirektion, Amt für Landwirtschaft. Vernetzungsperimeter. | Amt für Landwirtschaft: landwirtschaft@nw.ch |
| OW |  | Kanton Obwalden. Vernetzungsprojekte Obwalden. https://www.ow.ch/dienstleistungen/8036 (accessed 18 November 2024) | Dataset created using information from the given reference with municipal data from swisstopo. |
| SG |  | Kanton St.Gallen, Amt für Raumentwicklung und Geoinformation. 31-SG Perimeter Vernetzungsprojekte nach DZV. Datenkatalog. 2019. https://metadata.geo.sg.ch/geobasisdaten_rechtlich/352 (accessed 9 July 2025) | The dataset is available upon request from the Office for Spatial Development and Geoinformation. |
| SH |  | Kanton Schaffhausen, Landwirtschaftsamt Kanton Schaffhausen. Vernetzungsperimeter. https://opendata.swiss/de/dataset/vernetzungsprojekte-kanton-schaffhausen (accessed 18 November 2024) | Landwirtschaftsamt Kanton Schaffhausen:  la-sh@sh.ch |
| SO |  | Kanton Solothurn, Volkswirtschaftsdepartement, Amt für Landwirtschaft. Vernetzungsperimeter. | Amt für Landwirtschaft: alw.info@vd.so.ch |
| TG |  | Kanton Thurgau, Amt für Raumentwicklung. Vernetzungskorridore. 2003. https://shop.geo.tg.ch/products/vernetzungskorridore (accessed 11 November 2024) |  |
| TI |  | Repubblica e Cantone Ticino,  Dipartimento delle finanze e dell’economia, Divisione dell’economia, Sezione dell’agricoltura, Ufficio dei pagamenti diretti. IC Perimetri 2024. | Sezione dell’agricoltura: dfe-sa@ti.ch |
| UR |  | Kanton Uri, Amt für Landwirtschaft Kanton Uri. Vernetzungsperimeter. | Amt für Raumentwicklung: raumplanung@ur.ch |
| VD |  | Kanton Waadt, Departement für Finanzen und Landwirtschaft Kanton Waadt, Abteilung Direktzahlungen und Landwirtschafts- und Weinbaudaten. Vernetzungsperimeter. | Direction générale de l’agriculture, de la viticulture et des affaires vétérinaires (DGAV) : info.dgav@vd.ch |
| VS |  | Kanton Wallis, Dienststelle für Landwirtschaft, Amt für Direktzahlungen Kanton Wallis. Vernetzungsperimeter. | Amt für Direktzahlungen: sca-opd@admin.vs.ch |
| ZG |  | GIS Kanton Zug. Perimeter Vernetzungsprojekte VP. 2024. <https://zugmap.ch/bmcl/?project=ZugMap.ch&legend=alle%20Themen&rotation=0.00&scale=430054&center=2687339,1225039> (accessed 26 November 2024) |  |
| ZH |  | Kanton Zürich, Amt für Landschaft und Natur Kanton Zürich, Fachstelle Naturschutz. Vernetzungsperimeter. | Amt für Landschaft und Natur - Fachstelle Naturschutz:  naturschutz@bd.zh.ch |
| Swisstopo |  | Bundesamt für Landestopografie swisstopo. swissBOUNDARIES3D. 2024. https://www.swisstopo.admin.ch/de/landschaftsmodell-swissboundaries3d (accessed 26 November 2024) |  |
| FOAG |  | Federal Office for Agriculture. Landwirtschaftliche Zonengrenzen der Schweiz. 2024. https://data.geo.admin.ch/browser/index.html#/collections/ch.blw.landwirtschaftliche-zonengrenzen/items/landwirtschaftliche-zonengrenzen (accessed 26 November 2024) |  |

**Table A.2: Manual adjustments to data**

| Canton | Adjustment | Description | Code Reference |
| --- | --- | --- | --- |
| SH | Name harmonization | Several project names in ABS_info_SH manually reformatted with spaces/hyphens for consistency (e.g., 'Beringen-Löhningen-Neuhausen' → 'Beringen - Löhningen - Neuhausen'). | mutate(Name des Vernetzungsprojekt = if_else(...)) |
| SH | Text cleanup | Municipality names manually standardized ('Teile von' → 'parts of', 'Teil von' → 'part of', 'inkl.' → 'incl.'). | str_replace(Gemeinden, ...) |
| GL | Geometry correction | Projects incorrectly crossing community borders restricted using intersections: 'Hirzli', 'Kerenzerberg', 'Obersee-Schwändital' → Glarus Nord; 'Glarus' → Glarus; 'Glarus Süd' → Glarus Süd. | st_intersection(...) |
| JU | End year override | Remark field explicitly records that end_year was changed from original value. | mutate(remark = paste(..., 'end_year was changed...')) |
| JU | Community-specific flag | Project participation manually marked for 'Mervelier' (part_comm = 1). | mutate(part_comm = if_else(comm == 'Mervelier', 1, 0)) |
| LU | Spelling correction | 'Doppelschwand' corrected to 'Doppleschwand' in VZP_NAME. | mutate(VZP_NAME = if_else(...)) |
| LU | Community-specific flag | 'Luzern' community manually flagged (part_comm = 1). | mutate(part_comm = if_else(comm == 'Luzern', 1, 0)) |
| UR | End year override | end_year overwritten to 2027; remark field documents original end_year. | mutate(end_year = 2027, remark = paste(...)) |
| VD | Name harmonization | Several network names manually adjusted for merge consistency (e.g., 'Aubonne- La Côte' → 'Aubonne - La Côte'). | mutate(Nom_Réseau = if_else(...)) |
| VD | Canton reassignment | Projects in 'Courgevaux', 'Greng', 'Murten' reassigned from VD to FR. | mutate(canton = if_else(comm == ..., 'FR', 'VD')) |
| VD | End year override | Manual recalculation of end_year with remarks documenting change. | mutate(end_year = 2027, remark = paste(...)) |
| NE | Geometry union | 'EcoRéseau Joux-du-Plâne' geometry manually extended by unioning community geometries from BE (Renan, Sonvilier). | st_union(st_polygon(...)) |

1. **Additional information**

**When municipalities are considered *partial* ecological network projects**

The share of the ecological network projects area on the total possible network area is calculated by removing summering, lake and overlapping areas from the community area (leaving only the sum of ecological network projects implemented/not implemented areas). This share has a threshold value of 2.88%, too, which means that a municipality is considered a community with partially implemented ecological network projects, i.e. *part_comm* = 1, if between 2.88% and 97.12% of the total municipality area is part of a network project. If more (>97.12%) or less than this percentage (<2.88%) municipality is part of a network project, the municipality is shown as having fully ecological network projects implemented or no ecological network projects implemented, i.e. *part_comm* = 0.

We choose this threshold, as the municipality “Lupfig” (Canton of Aargau) has the smallest established ecological network project on its municipality area that we know with certainty, covering 2.88% of its municipality area. As examples, we plot the implemented and not implemented ecological network projects (ABS) areas for the municipalities of Lupfig (AG), Stein (AG) and Renens (VD) in the first row of Fig. A.1. We also provide the share of the implemented area out of the total municipality area in the top right boxes. These three municipalities are all considered municipalities with partially implemented ecological network projects (*part_comm* = 1).

For the Bodensee area (SG) and Göschenen (UR) municipality in the second row of Fig. A.1., we provide the share of the ecological network projects-implemented area out of the possible network area on the top right of the graph. At the top left, we provide the share of the implemented area of the total municipality area. These two examples are not considered municipalities with partially implemented ecological network projects, i.e. *part_comm = 0*. For Bodensee (SG), the reason is that the share of the ecological network projects-implemented area out of the total municipality area is only 1.2%, as lakes take up the largest share of the municipality. In Göschenen (UR), the implemented ecological network project covers 3.14% of the total municipal area, which exceeds the 2.88% threshold. However, over 97.12% of the potential network area is already utilized by these projects, as the remaining land is designated as summering area.


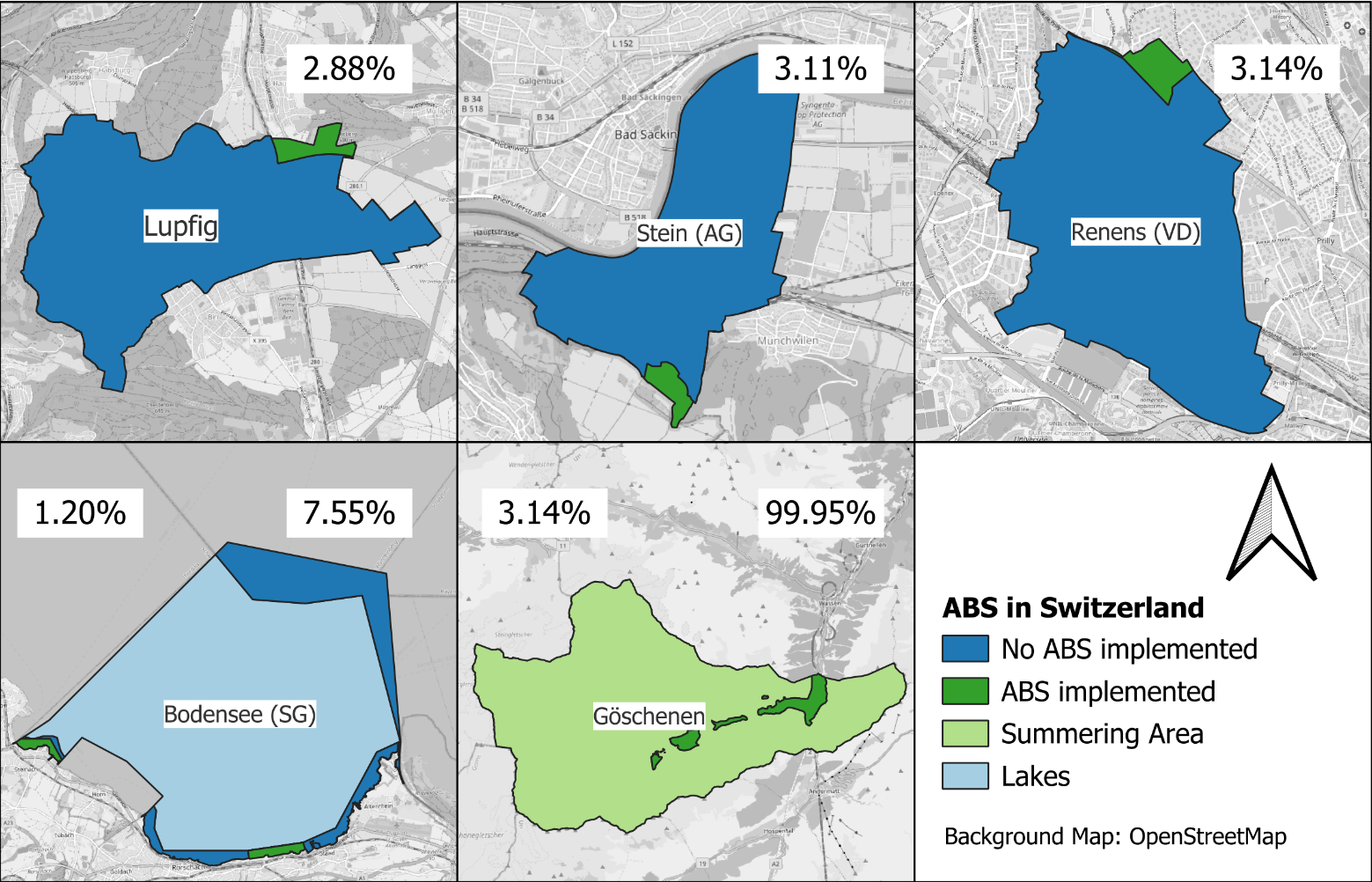


Network Projects (NP)

Lakes

Summering area

NP implemented

No NP implemented

Fig. A.1: This figure shows five municipalities and their ABS-implemented (green) and no ABS-implemented (blue) areas. In addition, the summering areas (light green) and lake areas (light blue) are visible. For the upper three municipalities, the percentage of the ABS-implemented area out of the total area is given. For the bottom two, for which the part_comm column equals 0, the percentage of the ABS-implemented area out of the total area is given in the left box and the share of ABS of the pure possible network area is shown in the right box.
